# Eye tracking evidence for the reinstatement of emotionally negative and neutral memories

**DOI:** 10.1371/journal.pone.0303755

**Published:** 2024-05-17

**Authors:** Paula P. Brooks, Brigitte A. Guzman, Elizabeth A. Kensinger, Kenneth A. Norman, Maureen Ritchey

**Affiliations:** 1 Princeton Neuroscience Institute, Princeton University, Princeton, NJ, United States of America; 2 Department of Psychology and Neuroscience, Boston College, Chestnut Hill, MA, United States of America; 3 Department of Psychology, Princeton University, Princeton, NJ, United States of America; Tilburg University, NETHERLANDS

## Abstract

Recent eye tracking studies have linked gaze reinstatement—when eye movements from encoding are reinstated during retrieval—with memory performance. In this study, we investigated whether gaze reinstatement is influenced by the affective salience of information stored in memory, using an adaptation of the emotion-induced memory trade-off paradigm. Participants learned word-scene pairs, where scenes were composed of negative or neutral objects located on the left or right side of neutral backgrounds. This allowed us to measure gaze reinstatement during scene memory tests based on whether people looked at the side of the screen where the object had been located. Across two experiments, we behaviorally replicated the emotion-induced memory trade-off effect, in that negative object memory was better than neutral object memory at the expense of background memory. Furthermore, we found evidence that gaze reinstatement was related to recognition memory for the object and background scene components. This effect was generally comparable for negative and neutral memories, although the effects of valence varied somewhat between the two experiments. Together, these findings suggest that gaze reinstatement occurs independently of the processes contributing to the emotion-induced memory trade-off effect.

## Introduction

*Watson: “Do you mean to say that you read my train of thoughts from my features?”*

*Holmes: “Your features, and especially your eyes.”*

**— Sir Arthur Conan Doyle**

(The Memoirs of Sherlock Holmes: *The Adventure of the Resident Patient*)

The idea of the eyes being a window into our thoughts is not a new one. In *The Adventure of the Resident Patient*, written in 1893, Mr. Sherlock Holmes is able to recount to his friend, Dr. John Watson, the details of Watson’s reverie based on where Watson had placed his eyes during his daydream. Indeed, recent scientific evidence suggests that we can infer people’s remembering based on eye movement patterns in a variety of contexts [1, 2]. During the initial viewing of an image, eye movements can predict whether or not the image will be subsequently remembered [3–6], above and beyond what would be predicted by the intrinsic properties of the image [4, 5]. Eye movement patterns during recognition are also sensitive to the contents of memory [5, 7]. In one study, researchers found that there were more eye fixations in the critical region where a previously studied scene had been altered, but only in participants without memory impairments [8]. Other studies have shown that eye movements during retrieval recapitulate the same pattern of eye movements during encoding [9–15], and this form of gaze reinstatement has been argued to actively facilitate memory retrieval through context reinstatement [16]. Put together, this suggests a link between eye movement patterns and memory performance.

An open question is whether gaze reinstatement is influenced by the affective salience of information stored in memory. Emotions have a significant impact on what we do and do not remember [17–19], leading to biases in memory toward affectively salient information [18, 20]. Such biases have been clearly demonstrated in studies of the emotion-induced memory trade-off effect, where memory for negative objects is better than that for neutral objects, but at the expense of remembering the associated neutral background [21]. Furthermore, negative emotion might actually increase the reinstatement of perceptual details [22], suggesting that retrieval of visual information, and its associated eye movements, may differ for negative compared to neutral stimuli. Affectively salient information has been shown to attract visual attention, characterized by fixation patterns during perception [23, 24]. However, these changes in attention during encoding do not appear to explain the emotion-induced memory trade-off effect [25], leaving open the possibility that retrieval processes play a role in generating this effect.

The goal of our study was to investigate whether eye movement patterns during retrieval are biased toward the remembered location of affectively salient information. To do this, we optimized the emotion-induced memory trade-off paradigm for use with eye tracking. Importantly, our stimuli were carefully created so that objects were either located on the left or right side of the scene. This allowed us to evaluate gaze reinstatement by measuring whether the participant was looking at the corresponding side of the screen during retrieval, when the object was no longer shown. Based on the prior work reviewed above, we hypothesized that gaze reinstatement would be greater during the retrieval of affectively negative compared to neutral information, in what we believe to be the first study investigating whether retrieval-related eye movement patterns are sensitive to emotional memory biases.

## Experiment 1

### Methods

#### Participants

Thirty-four participants from Boston College participated in this experiment. Participants were 18–35 years old, had normal to corrected-to-normal color vision, were native speakers of English since early childhood, and self-reported to be free of any neurological or psychiatric disorders. Data from 6 participants were excluded because of an excess of missing eye tracker data and 4 participants were unable to complete the study because of computer issues. In total, data from 24 participants (9 male, 15 female; age = 18–22 years, mean = 19.3 years) were included in our analyses. This experiment was conducted with the approval of the Institutional Review Board (IRB) at Boston College with an IRB Authorization Agreement from Princeton University. After considering at the sample sizes of other eye tracking studies like [9, 26], which analyzed data from 17 and 19 participants respectively, we determined that it would be feasible to collect and analyze data from 24 participants within a reasonable time frame. Recruitment took place between January 24, 2022 and September 13, 2022. All participants provided written informed consent prior to participation.

#### Stimuli

Stimuli consisted of 6 sets of 15 object-background scenes, where each background could be associated with either a negative or neutral object on the left or right side of the scene. Images were assigned to object valence and object location conditions using a Latin-square counterbalancing design that included 6 sets (see Section A in S1 Text for complete details). This meant that any stimulus characteristics of the background scene or its pairing with the foreground object should not systematically bias our results. Furthermore, we aimed to make the pairings of foreground objects and background scenes semantically congruent so that they could be meaningfully related to each other. Finally, stimuli were selected in such a way so that they reflected distinct semantic concepts. In total, each participant studied 60 scene images (object valence: 30 negative, 30 neutral; object location: 30 left, 30 right). They also viewed 30 new backgrounds and 30 new objects (15 negative, 15 neutral) that were used as non-similar foils during the final memory tests. Finally, 12 additional object-background scenes were used for practice. The stimuli were from [21], as well as from the internet.

A separate norming study was conducted online to assess the valence and arousal ratings of both individual objects and composite scene images (Table 1). Forty participants rated 192 individual objects (180 critical, 12 practice) along with 96 composite scenes (90 critical, 6 practice; equally divided in object valence and location conditions). One-way ANOVAs revealed that there was a statistically significant difference in mean object valence and arousal between negative and neutral objects (*F*(1,78) = 406.51, *p* < 0.001 and *F*(1,78) = 152.61, *p* < 0.001, respectively). Furthermore, there was a statistically significant difference in mean scene valence and arousal between negative and neutral scenes (*F*(1,78) = 335.28, *p* < 0.001 and *F*(1,78) = 88.65, *p* < 0.001, respectively).

**Table 1:** Descriptive results from stimulus norming study: Means and standard deviations of valence and arousal ratings for object (N = 40) and scene (N = 10) images. Significance between negative and neutral items was calculated using one-way ANOVAs.

	Negative		Neutral			
**Object Ratings**	Mean	Std	Mean	Std	*F*(df)	p-value
Valence	2.999	0.531	5.510	0.582	406.51 (1, 78)	< 0.0001
Arousal	6.233	0.568	4.368	0.768	152.61 (1, 78)	< 0.0001
	Negative		Neutral			
**Scene Ratings**	Mean	Std	Mean	Std	*F*(df)	p-value
Valence	3.259	0.553	5.498	0.541	335.28 (1, 78)	< 0.0001
Arousal	6.077	0.698	4.432	0.857	88.65 (1, 78)	< 0.0001

#### Procedure

**Fig 1:**
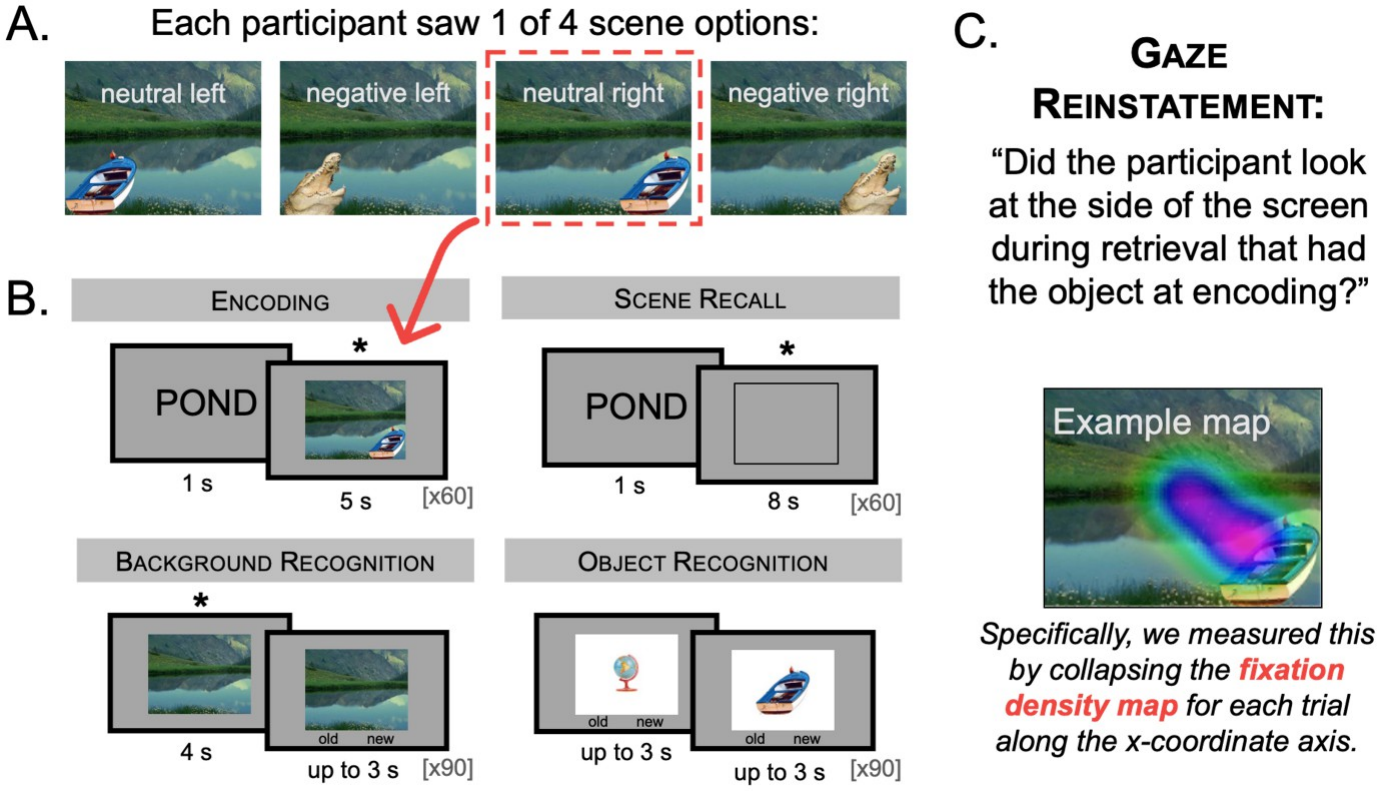
Schematic of experiment design. (A) Object-background scene images were used in this experiment. Each background could be associated with either a negative or neutral object positioned on the left or the right of the scene. The scenes were counterbalanced across participants so that each participant only saw one of the four possible scene options. (B) In this eye tracking experiment, participants studied word-scene pairs before doing a short distractor odd/even task (not shown). Participants then did a scene recall task, during which they were shown the cue word and had to vividly bring the associated scene to mind within the confines of an empty box. Finally, participants completed separate background and object recognition tasks. Importantly, there was a brief waiting period during the background recognition task where the background image appeared on the screen but the participant had to wait 4 seconds before being able to respond. The asterisks denote portions of particular interest to us for our eye tracking analyses. (C) We measured gaze reinstatement in terms of whether people looked more at the side of the screen at retrieval that had the object during encoding (e.g., whether their eye movement patterns could discriminate between left and right trials).

Participants completed a modified emotion-induced memory trade-off paradigm task during a single experiment session (Fig 1). Eye tracking data were collected throughout the entire experiment and a 5-point calibration/validation procedure was done before every phase.

Participants first completed the encoding phase, where they studied 60 critical word-scene pairs. Each word was semantically related to its associated scene, which was composed of a negative or neutral object superimposed on the left or right of a neutral background (counterbalanced conditions: 15 negative left, 15 negative right, 15 neutral left, and 15 neutral right). At the start of each trial (inter-trial interval, or ITI = 2 s), each word appeared on the screen for 1 s. After this, the scene appeared on the screen for 5 s followed by a prompt for participants to use the keyboard to indicate whether they would (1) approach, (2) stay the same distance away, or (3) back away from the scene if they encountered it in real life. Participants were told to associate the words to the scenes so that they could vividly visualize the scene when shown just the word. Participants saw each word-scene pair one time.

Participants then completed the distractor phase during which numbers (1–99) appeared on the screen, one at a time, and the participants had to use the keyboard to say whether the number was odd or even. Participants had 45 s to complete as many trials as they could, in a self-paced fashion.

Participants then completed the scene recall phase where they were then shown each word for 1 s (ITI = 1 s) and given the task of bringing the associated scene vividly to mind for 8 s, selected to match the period used in [26]. During this time, participants were told to vividly visualize the associated scene as accurately and in as much detail as possible within the confines of an empty box that appeared on the screen. This box was the same size as and in the same location as the scenes studied at the start of the experiment, as in [26]. Participants then had up to 2 s to indicate how vividly they were able to visualize the scene using a 4-point scale (1 = forgot, 2 = very hazy, 3 = in the middle, 4 = very vivid).

Participants then completed the background recognition phase with 60 old backgrounds and 30 new backgrounds. The background first appeared on the screen by itself for 4 s (ITI = 1 s), during which time participants had to think about whether or not they previously saw it. Then, a scale appeared at the bottom of the screen and participants had up to 3 s to indicate whether the background was “old” (included in a previously studied scene) or “new” (not previously studied).

Finally, participants completed the object recognition phase with 60 old objects and 30 new objects. Participants had up to 3 s to indicate whether each object was “old” or “new” (ITI = 1 s). To be clear, there was no “waiting” period during this phase—participants could indicate their answer as quickly as they wanted.

#### Data acquisition and preprocessing

The experiment task was programmed using MATLAB Psychtoolbox and a Tobii Pro Spectrum eye tracker was used to record eye movements at a sampling rate of 120 Hz, with a screen resolution of 1920 by 1080. Participants sat approximately 63 cm away from the screen, but the distance was recorded continuously throughout the experiment. We collected gaze data for every trial of the experiment. Data were collected using the Tobii SDK MATLAB toolbox and a standard 5-point calibration/validation was carried out before each task phase. Self-reported questionnaires, including the Positive and Negative Affect Schedule (PANAS; [27]) and the State-Trait Anxiety Inventory (STAI; [28]), were administered using REDCap electronic data capture tools hosted at Boston College [29].

Eye tracking data were preprocessed as in [30]. To summarize, we began by averaging x-, y-, and z-coordinate values for the left and right eyes to get a single value for eye movements. We then smoothed the eye movement values to reduce the noise, using the median of a 5-point overlapping sampling window. Next, we transformed the coordinates from Tobii Pro space (Active Display Coordinate System) to pixel space, with the origin being at the top left corner of the screen. This allowed us to more easily reference eye movement patterns to where the stimuli appeared on the screen. After this, we identified valid trials, or trials for which data did not fall outside of the screen more than 60% of the time. We only analyzed data from valid trials and we used the average z-coordinate value, calculated separately for every trial, to compute the visual angle. We then identified fixations, whose samples could not be more than 1 degree of a visual angle apart and be at least 100 ms long [1], using a dispersion-threshold identification algorithm [31]. We calculated the dispersion of the minimum number of time points needed for a fixation and kept adding samples to it if the dispersion did not surpass the pixel threshold corresponding to 1 degree of the visual angle. Finally, we cleaned fixations by merging fixations if their centers were within 0.5 degrees of a visual angle and were less than 75 ms apart.

#### Analysis methods

Memory accuracy was calculated by using hits minus false alarms. A factorial repeated measures ANOVA, with the object valence and scene component (object or background) as the factors, was used to calculate significance. The effect size was measured using Cohen’s *d*_*t*_, which was calculated using the *t*-statistic over the square root of the sample size [32, 33].

Fixation density maps were created for every trial, using a procedure similar to [16]. Specifically, we used two-dimensional kernel density estimation as implemented in the KernelDensity function from the Python scikit-learn software library [34]. We computed the kernel density of each point (*x,y*) on a uniform grid (width = 70, height = 55) spanning the dimensions of the presented images (700 by 550 pixels) by using a Gaussian kernel and setting the bandwidth to 80 pixels. To be clear, this means that our analyses only considered fixations that fell on the stimulus boundary (e.g., where the empty box or scene image appeared on the screen). Finally, fixations were normalized over space.

We measured gaze reinstatement by computing the average of the duration-weighted fixation density maps, which were calculated as in [35], along the x-coordinate axis for trials where the object was on the left versus the right, and then measuring the separation of these weighted average values on left versus right trials using area under the Receiver Operating Characteristic curve (AUC). We were able to take this approach because of the unique characteristic of our stimulus images having objects either on the left or the right of the scene. Specifically, we resampled our data with replacement and calculated an AUC value 1000 times on the combined individual trial data of all the participants into a single “supersubject”, as described in [36]. This bootstrapping approach did not lend itself to a standard effect size calculation, so none have been reported for these analyses. The p-value was calculated by computing 1 minus [the number of bootstrap instances that produced an AUC value that was above chance (or 0.5) / total number of bootstraps (1000)]. We also took the difference between AUC values, in which case the p-value was calculated using 0 as chance. The gaze reinstatement comparisons between correct versus incorrect object memory and correct versus incorrect background memory were restricted to old items.

Finally, our analysis results were plotted using seaborn and pandas Python packages. We used statannot to add statistical annotations on our plots, using paired t-tests and Bonferroni correction unless otherwise mentioned. We used the function rm_anova from the Python package pingouin to compute our factorial repeated measures ANOVAs.

### Results

#### Memory performance

**Fig 2:**
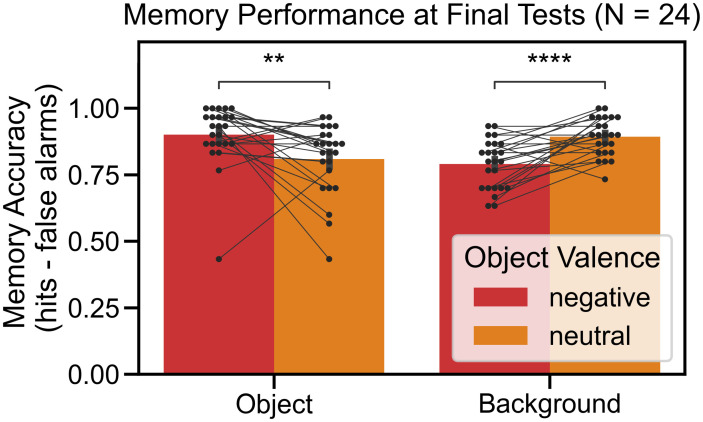
Experiment 1: Emotion-induced memory trade-off effect. There was a significant interaction between object valence and scene component. Negative objects were remembered better than neutral ones but at the expense of remembering the associated background. All error bars denote standard error of the mean. Paired comparisons were performed using paired t-tests with Bonferroni correction, ** *p* < 0.01, **** *p* < 0.0001.

We replicated the emotion-induced memory trade-off effect (Fig 2). Memory accuracy was entered into a repeated measures ANOVA with the object valence (negative, neutral) and scene component (object, background) as within-subjects factors. There was no main effect of object valence, *F*(1, 23) = 0.166, *p* = 0.687, $\eta_{p}^{2}=0.007$, nor was there a main effect of scene component, *F*(1, 23) = 0.378, *p* = 0.545, $\eta_{p}^{2}=0.016$. However, there was a significant interaction between object valence and scene component, *F*(1, 23) = 36.449, *p* < 0.001, $\eta_{p}^{2}=0.613$. There was higher memory accuracy for negative objects than for neutral objects, *t*(23) = 4.034, *p* = 0.001, *d*_*t*_ = 0.823, but at the expense of the associated background memory. Background memory for associated negative objects was lower than for associated neutral objects, *t*(23) = 5.314, *p* < 0.001, *d*_*t*_ = 1.085. The emotion-induced memory trade-off effect was driven by differences in hit rates; please refer to Section B in S1 Text to look at final test performance by hit and false alarm rates. Moreover, we did not find a memory bias for items appearing in the left or the right object location; please refer to Section C in S1 Text for additional details.

#### Eye movements during encoding

**Fig 3:**
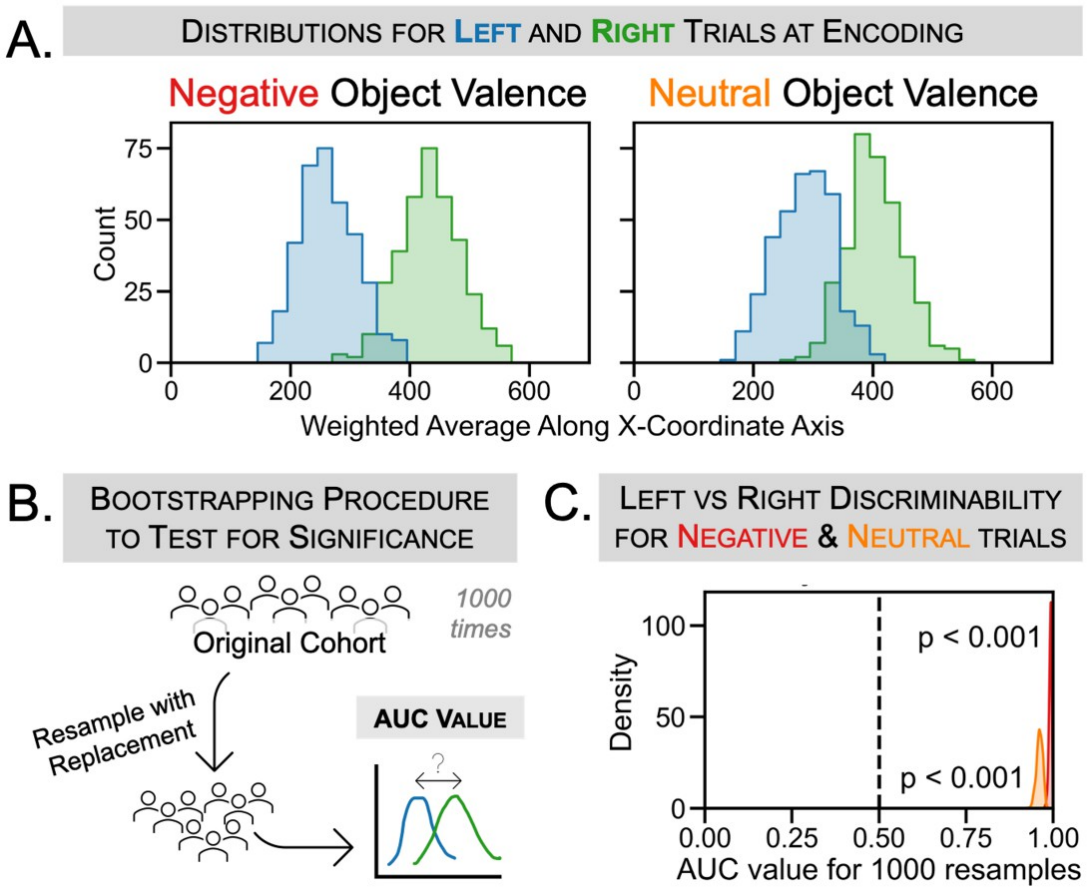
Experiment 1: Gaze reinstatement shown at encoding. At encoding, people looked toward the object location (left or right), especially when the object was negative. (A) Here we show a histogram of the weighted averages of the fixation density maps collapsed along the x-coordinate axis. Each histogram, for negative and neutral valence trials separately, shows two distributions: one for trials where the object was on the left (blue) and the other for the right (green). (B) To verify that left and right fixations were significantly different, we resampled our data and calculated an AUC value 1000 times. (C) The left and right distributions were highly discriminable at encoding for both negative (red) and neutral (orange) object valence trials.

Turning to the eye movement data, we first sought to confirm that our approach could distinguish between eye movements to objects placed on the left versus right side of the screen. Indeed, during the encoding phase, participants tended to look toward the object location (left or right). This preference toward the side with the object was especially true when the object was negative compared to when it was neutral (Fig 3), indicating a bias in attention toward negative stimuli. The mean AUC value for negative object valence was 0.992 (*p* < 0.001) and for neutral object valence was 0.961 (*p* < 0.001); the difference in these AUC values was significant (*p* < 0.001).

#### Gaze reinstatement during scene recall and background recognition

**Fig 4:**
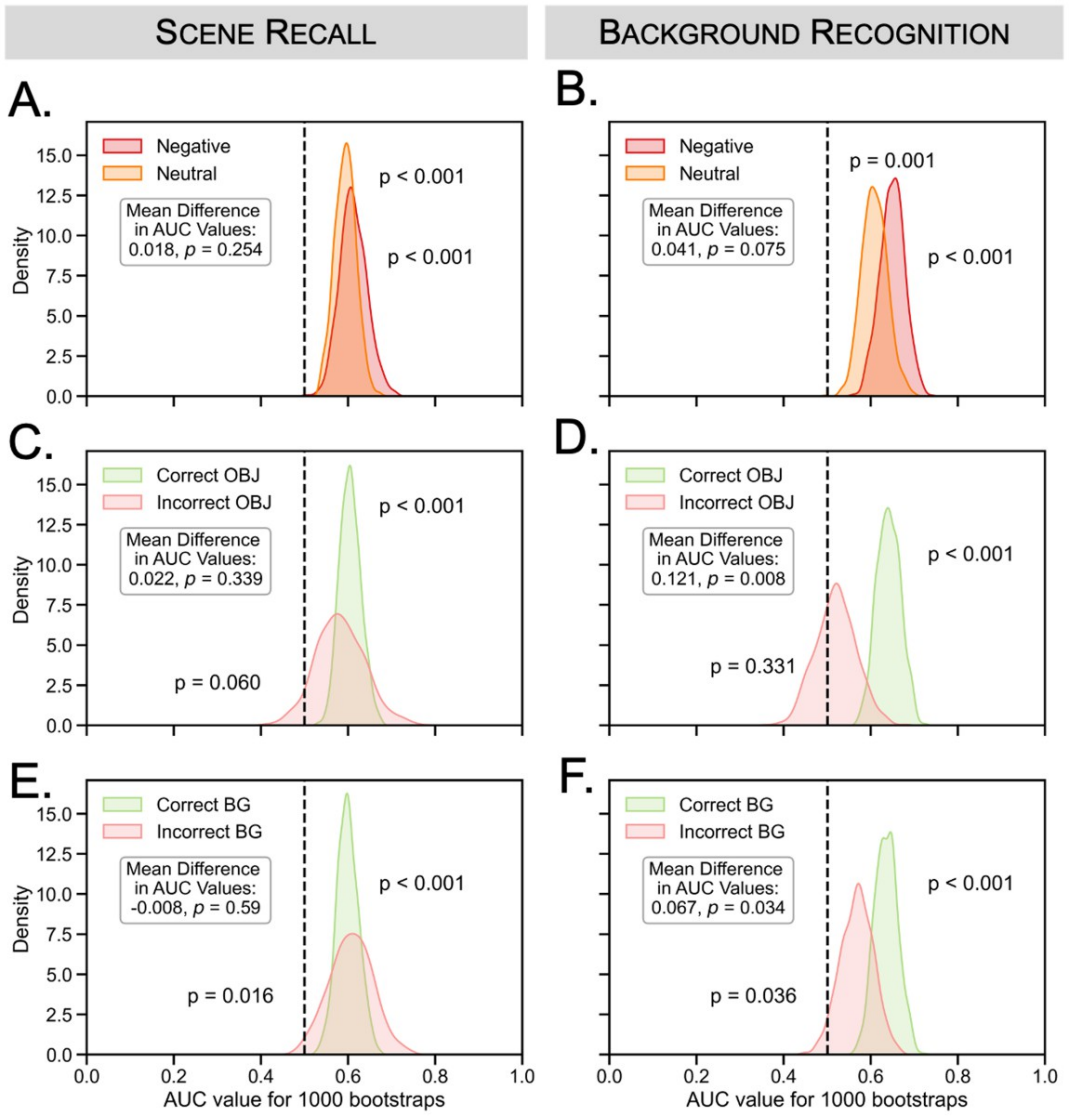
Experiment 1: Eye tracking results by final test memory and object valence. The figure shows the distributions of AUC values for negative (red) and neutral (orange) trials during (A) scene recall and (B) background recognition. There was no significant difference in AUC values between negative and neutral trials during the scene recall or background recognition phases. AUC distributions are also shown for correct (green) versus incorrect (red) (C, D) object and (E, F) background memory for scene recall and background recognition, respectively. There was more gaze reinstatement for correct versus incorrect object memory in the background recognition phase but not the scene recall phase. We found similar results for correct versus incorrect background memory.

Next we tested whether gaze reinstatement during the scene recall phase and background recognition phase was modulated by object valence and memory accuracy (Fig 4). During the scene recall phase, there was evidence of gaze reinstatement for both negative (*p* < 0.001) and neutral trials (*p* < 0.001), but there was no significant difference between the two valence conditions. The mean difference in AUC values for negative versus neutral objects was 0.018, *p* = 0.254. Similarly, during background recognition, there was evidence of gaze reinstatement for both negative (*p* < 0.001) and neutral trials (*p* = 0.001), but there was no significant difference between the two object valence conditions (mean difference = 0.041, *p* = 0.075). In other words, we did not find a significant main effect of object valence on gaze reinstatement during scene recall or background recognition. Turning to the comparisons between correct and incorrect memory, we found an effect of recognition memory on gaze reinstatement during background recognition, but not the scene recall phase. During background recognition, there was more gaze reinstatement for trials associated with correct versus incorrect object memory (mean difference = 0.121, *p* = 0.008) but there was no such difference for the scene recall phase (mean difference = 0.022, *p* = 0.339). Similarly, there was more gaze reinstatement for trials associated with correct versus incorrect background memory during background recognition (mean difference = 0.067, *p* = 0.034) but not during the scene recall phase (mean difference = -0.008, *p* = 0.590).

#### Interaction between memory and valence during background recognition

**Fig 5:**
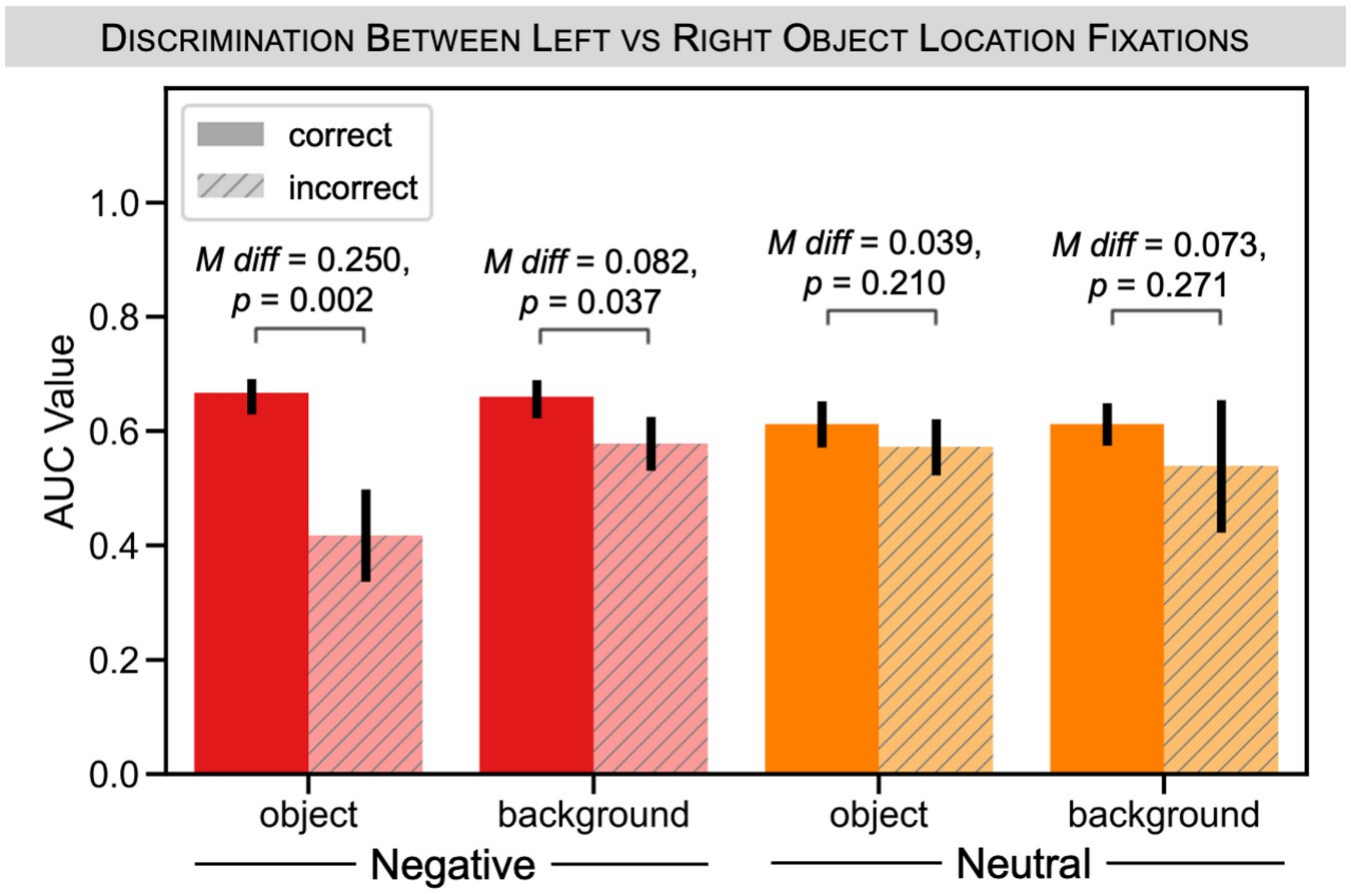
Experiment 1: Gaze reinstatement results split by valence and memory. We looked at the separability of fixations associated with left versus right object locations during background recognition by object valence and final test performance. The figure depicts AUC values that quantify the separation of the weighted average values for left versus right object trials following our bootstrapping procedure, computed separately for negative (red) and neutral (orange) trials when the participant got the object and background correct (solid) versus incorrect (striped). For negative trials, there was a significant difference in AUC values between correct versus incorrect object memory, as well as for correct versus incorrect background memory. However, we saw no such difference between correct and incorrect object or background memory for neutral trials, resulting in a significant interaction between valence and memory for object memory but not background memory. All error bars denote standard deviation of the bootstrap.

Lastly, we tested whether the effect of recognition memory on gaze reinstatement was modulated by valence, focusing on the background recognition phase in which we had observed the memory effect. Specifically, we tested whether there was an interaction between recognition memory and object valence (Fig 5). For negative valence items, we found that gaze reinstatement was significantly modulated by object and background memory. There was a larger AUC value for trials associated with correct versus incorrect negative object memory (mean difference = 0.250, *p* = 0.002). There was a similar pattern for background memory (mean difference = 0.082, *p* = 0.037). However, for neutral valence items, AUC was not significantly modulated by object (mean difference = 0.039, *p* = 0.210) or background memory (mean difference = 0.073, *p* = 0.271). To test for the interaction, we computed the difference between the AUC scores during background recognition for trials associated with correct versus incorrect object or background memory separately for our two valence conditions; and we then computed the difference between negative versus neutral:

$$\begin{array}{c} \left(Correct_{negative}-Incorrect_{negative}\right)-\left(Correct_{neutral}-Incorrect_{neutral}\right) \end{array}$$ (1)

We found that these differences were significant for object memory (*p* = 0.013) but not for background memory (*p* = 0.463). In other words, gaze reinstatement was more strongly related to object memory for negative compared to neutral trials, while its relationship to background memory did not significantly differ between the valence conditions.

### Discussion

Do eye movements reveal the contents of people’s memories? And do they reveal memory biases toward emotional information? In this first experiment, we found evidence for gaze reinstatement in individuals’ eye movement patterns during both scene recall and background recognition, though only during background recognition did this measure track with memory performance. There was no main effect of valence on gaze reinstatement, but there was an interaction showing that gaze reinstatement was more related to object memory for negative compared to neutral trials. While prior work has examined the relation between eye gaze at encoding and memory for emotional and neutral objects paired with scenes [37, 38] to our knowledge, this is the first examination of how gaze reinstatement is modulated by affective salience and memory. Using the emotion-induced memory trade-off paradigm, we also replicated previous findings demonstrating that negative objects were remembered better than neutral objects at the expense of the associated background memory.

It is worth noting that our approach for calculating gaze reinstatement was novel compared to what other people have done previously. Often, gaze reinstatement is computed by calculating the Pearson’s correlation between the fixation density map produced at encoding and retrieval, while taking different measures to control for individual differences in viewing patterns. This method, however, can be complicated by individual differences in the dispersion of fixations when an image is being imagined, like it was done in the scene recall phase, than when it is perceived, like in the encoding phase [39, 40]. This contributed to why Bone and colleagues [26] aligned the encoding and retrieval fixation maps using an orthogonal Procrustes transformation to minimize the distance between the two fixation maps before calculating the Pearson’s correlation in their study. To avoid these complicating factors, we developed our simpler approach, leveraging the unique characteristics of our stimuli (specifically, the left- and rightness of the objects) to assess gaze reinstatement as the discrimination between gaze patterns associated with left- versus right-associated scenes. The novelty of this approach warranted a replication of this study, which we undertook in Experiment 2. We also took this as an opportunity to refine our experiment design and to include an explicit memory test for object location.

## Experiment 2

In this experiment, we sought to corroborate the results from our modified emotion-induced memory trade-off paradigm. Specifically, we wanted to validate our novel approach for measuring gaze reinstatement. This experiment was collected a year after Experiment 1 in an independent cohort of participants, and our methods and predictions were preregistered on the Open Science Framework (https://osf.io/uh7y5/), following the methods and results reported for Experiment 1.

### Methods

#### Participants

Twenty-seven participants from Boston College participated in this experiment. Participants were 18–35 years old, had normal to corrected-to-normal color vision, were native speakers of English since early childhood, and self-reported to be free of any neurological or psychiatric disorders. Data from two participants were excluded because of too much missing data during a critical part of our analyses (the background recognition phase), and data from one participant was excluded because one experiment phase was omitted due to experimenter error. In total, data from 24 participants (9 male, 15 female; age = 18—22 years, mean = 19.6 years) were included in our analyses, targeting the same final sample size as in Experiment 1. This experiment was conducted with the approval of the Institutional Review Boards at Boston College with an IRB Authorization Agreement from Princeton University. Recruitment took place between January 25, 2023 and February 23, 2023. All participants provided written informed consent prior to participation.

#### Stimuli

We used the same object-background scene pairs as in Experiment 1. The only modification we made was that we took extra care to make sure that all the objects were the same distance away from the center, regardless of the location condition. Furthermore, we made sure that all of the objects associated with a given background were located at the same height along the y-coordinate axis. All changes were made using Adobe Photoshop.

#### Procedure

Participants completed the modified emotion-induced memory trade-off paradigm used in Experiment 1. There were only 2 modifications to the procedure. The first modification was based on our observation that there was no significant difference between the AUC values produced in the first half of the retrieval portion of the scene recall phase in Experiment 1 with those produced in the second half (*p* = 0.624 following the permutation test described above). As a reminder, participants were instructed to vividly visualize the associated scene as accurately and in as much detail as possible within the confines and duration of the empty box that appeared on the screen; these instructions might explain why there was no difference in the first and second half. As a result, we reduced the time participants spent vividly visualizing the scene during the scene recall phase from 8 to 4 s. We reduced this time to fit everything in our 1.5 hour study time slot while also adding another experiment phase to the end of the study. We incorporated a cued recall and location memory test phase at the very end of the experiment in order to better understand how eye movement patterns were related to memory for object identity and location. This task is described in more detail below.

At the very end, object memory was tested using a cued recall task, along with location memory. Participants had to type a written description of the associated object when presented with the background cue, and they also had to use the mouse to indicate where the center of the object had been located. The background appeared on the screen for 2 s and participants had up to 10 s to type their description. Participants could either press the RETURN key when they were ready to continue, or they were automatically taken to the next part if the time had run out. At this point, participants had up to 5 s to use the mouse to indicate the center of where the object had been located (ITI = 0.5 s).

#### Data acquisition and preprocessing

Data were acquired and preprocessed using the same methods as in Experiment 1.

#### Analysis methods

Background and object recognition accuracy were evaluated as in Experiment 1. We additionally considered performance on the object recall and location memory tests to corroborate our interpretation of the recognition results. In our preregistration, we had said that we would score location memory accuracy by whether or not the participant’s answer fell on the object mask (where the object had been located during encoding). However, performance was near floor with this approach, suggesting it may be too conservative of a criterion. As a result, we subsequently decided that it would be better for this measure to correspond with our eye tracking analysis approach, where we considered anything in the correct half of the screen (e.g., to the left of center along the x-coordinate axis for objects that were on the left) to be evidence for memory.

Two participants did not complete the last part of the experiment (cued recall and location memory test phase) because of scheduling issues. One participant completed the third phase of the experiment (scene recall phase) but the eye tracker did not properly record their eye data so we could not include that participant in our eye tracking analyses involving this portion of the experiment. An additional 3 participants did not follow instructions during the cued recall portion of the final memory test phase so their data was excluded from these analyses.

Finally, we also used the function AnovaRM from the Python package statsmodels to compute our factorial repeated measures ANOVA with three within-subjects factors.

### Results

#### Memory performance

**Fig 6:**
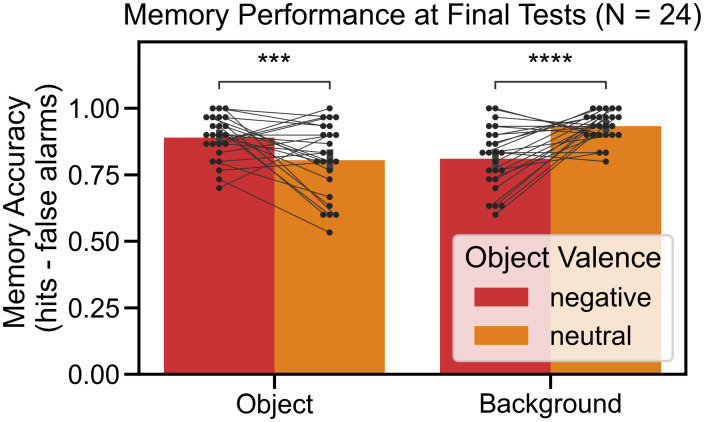
Experiment 2: Emotion-induced memory trade-off effect. The results replicated the emotion-induced memory trade-off effect. There was a significant interaction (p < 0.0001) between object valence and scene component. Negative objects were remembered better than neutral objects but at the expense of remembering the associated background. All error bars denote standard error of the mean. Statistics were performed using paired t-tests with Bonferroni correction, *** *p* < 0.001, **** *p* < 0.0001.

The recognition memory results replicated the results of Experiment 1, again showing the emotion-induced memory trade-off effect (Fig 6). Memory accuracy was entered into a repeated measures ANOVA with the object valence (negative, neutral) and scene component (object, background) as within-subjects factors. There was no main effect of object valence, *F*(1, 23) = 2.126, *p* = 0.158, $\eta_{p}^{2}=0.085$, nor was there a main effect of scene component, *F*(1, 23) = 1.823, *p* = 0.190, $\eta_{p}^{2}=0.073$. However, there was a significant interaction between object valence and scene component, *F*(1, 23) = 36.310, *p* < 0.001, $\eta_{p}^{2}=0.612$. There was higher memory accuracy for negative objects than for neutral objects, *t*(23) = 4.121, *p* < 0.001, *d*_*t*_ = 0.841, but at the expense of the associated background memory. Background memory for associated negative objects was lower than for associated neutral objects, *t*(23) = 5.478, *p* < 0.001, *d*_*t*_ = 1.118. Like in Experiment 1, the emotion-induced memory trade-off effect was driven by differences in hit rates, and we did not find a memory bias for items appearing in the left or the right object location; please refer to Sections B and C in S1 Text for additional details.

#### Eye movements during encoding

During the encoding phase, replicating the results of Experiment 1, we found that participants tended to look toward the object location (left or right), and this preference toward the side with the object was especially true when the object was negative. The mean AUC value for negative object valence was 0.990 (*p* < 0.001) and for neutral object valence was 0.962 (*p* < 0.001); the difference in these AUC values was significant (*p* < 0.001).

#### Gaze reinstatement during scene recall phase and background recognition

**Fig 7:**
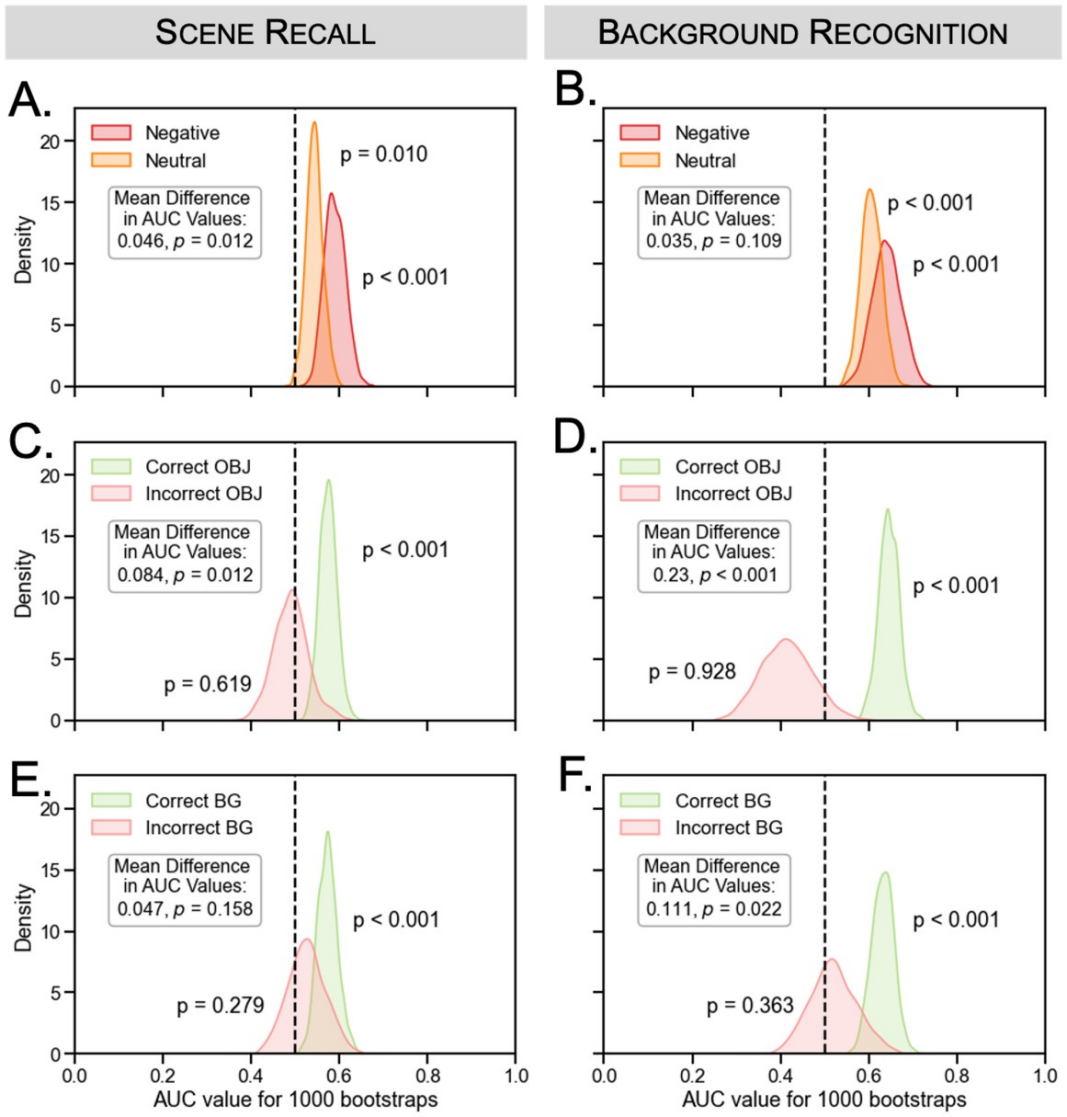
Experiment 2: Eye tracking results by final test memory and object valence. The figure shows the AUC distributions for negative (red) and neutral (orange) trials during (A) scene recall and (B) background recognition. There was a significant difference in AUC values between negative and neutral trials during scene recall but not background recognition. AUC distributions are also plotted for correct (green) versus incorrect (red) (C-D) object and (E-F) background memory during scene recall and background recognition, respectively. There was more gaze reinstatement for correct versus incorrect object memory for both phases; there was more gaze reinstatement for correct versus incorrect background memory in the background recognition phase, but not the scene recall phase.

Replicating the results of Experiment 1, there was evidence for gaze reinstatement during both scene recall (negative: *p* < 0.001, neutral: *p* = 0.010) and background recognition (negative: *p* < 0.001, neutral: *p* < 0.001) (Fig 7). As in Experiment 1, there was no effect of object valence on gaze reinstatement during background recognition (mean AUC difference = 0.035, *p* = 0.109), but surprisingly, there was a significant valence difference during scene recall (mean AUC difference = 0.046, *p* = 0.012), such that gaze reinstatement was greater for negative compared to neutral trials.

Replicating the results of Experiment 1, we also found evidence that gaze reinstatement during background recognition was related to memory accuracy. There was a significant difference between correct and incorrect object memory during background recognition (mean difference = 0.230, *p* < 0.001). Unlike in Experiment 1, there was also a significant effect of object memory in the scene recall phase (mean difference = 0.084, *p* = 0.012). As in Experiment 1, we found a difference between correct versus incorrect background memory during background recognition (mean difference = 0.111, *p* = 0.022) but not during scene recall (mean difference = 0.047, *p* = 0.158).

#### Interaction between memory and valence during background recognition

**Fig 8:**
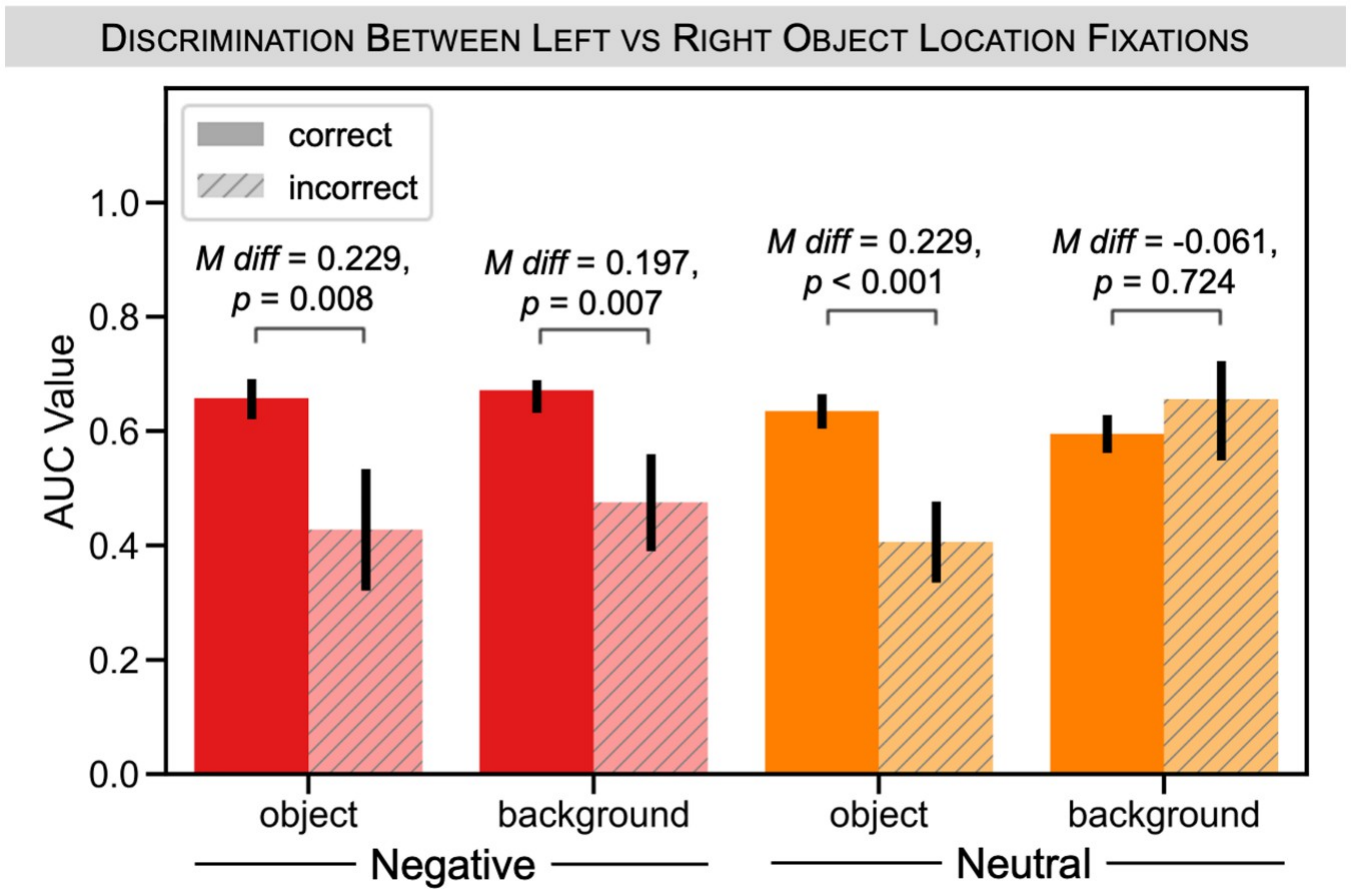
Experiment 2: Gaze reinstatement results split by valence and memory. We looked at the separability of fixations associated with left versus right object locations during background recognition by object valence and final test performance. We plotted AUC values that quantify the separation of the weighted average values for left versus right object trials following our bootstrapping procedure. We were particularly interested in the difference in AUC values for negative (red) and neutral (orange) trials when the participant got the object and background correct (solid) versus incorrect (striped). For negative trials, there was a significant difference in AUC values between correct versus incorrect object memory, as well as for correct versus incorrect background memory. For neutral trials, we saw a similar difference only for correct versus incorrect object memory, but not background memory. All error bars denote standard deviation of the bootstrap.

Finally, we sought to replicate the valence by memory interaction identified in Experiment 1. As in Experiment 1, for negative valence items, we found that AUC was modulated by object and background memory (Fig 8). There was a larger AUC value for correct versus incorrect negative object memory (mean difference = 0.229, *p* = 0.008). We found a similar pattern for background memory for negative trials (mean difference = 0.197, *p* = 0.007). However, unlike in Experiment 1, there was a comparable difference between correct versus incorrect object memory for neutral trials (mean difference = 0.229, *p* < 0.001), and no difference between correct versus incorrect background memory for neutral trials (mean difference = -0.061, *p* = 0.724). To quantify the interaction, we computed the AUC differences separately for object and background memory. Surprisingly, we observed a different pattern of interactions than in Experiment 1: here, there was not a significant interaction effect for object memory, *p* = 0.476, but there was a significant interaction for background memory, *p* = 0.020. In other words, background memory (expressed via recognition judgments) and gaze reinstatement appeared to be more strongly related for negative versus neutral trials, whereas object memory was similarly related to gaze reinstatement for both negative and neutral trials.

Given the fact that we saw two different patterns of valence by memory interactions in Experiment 1 (interaction for object memory) and Experiment 2 (interaction for background memory), we reran this same analysis using data from both experiments to determine which pattern of results would emerge across this combined sample (see Section D in S1 Text for complete details). In the combined sample (N = 47), we found that object memory was related to gaze reinstatement for both negative and neutral trials. In contrast, we found that background memory was related to gaze reinstatement for negative trials, but not the neutral trials. However, when we re-ran the interaction analyses, computing the AUC differences as before, we did not find a significant interaction effect for either object memory, *p* = 0.109, or for background memory, *p* = 0.112. Thus, although there were nonsignificant numerical trends in this direction, there was no consistent evidence that valence modulated the relationship between gaze reinstatement and recognition memory performance.

#### Location memory performance

**Fig 9:**
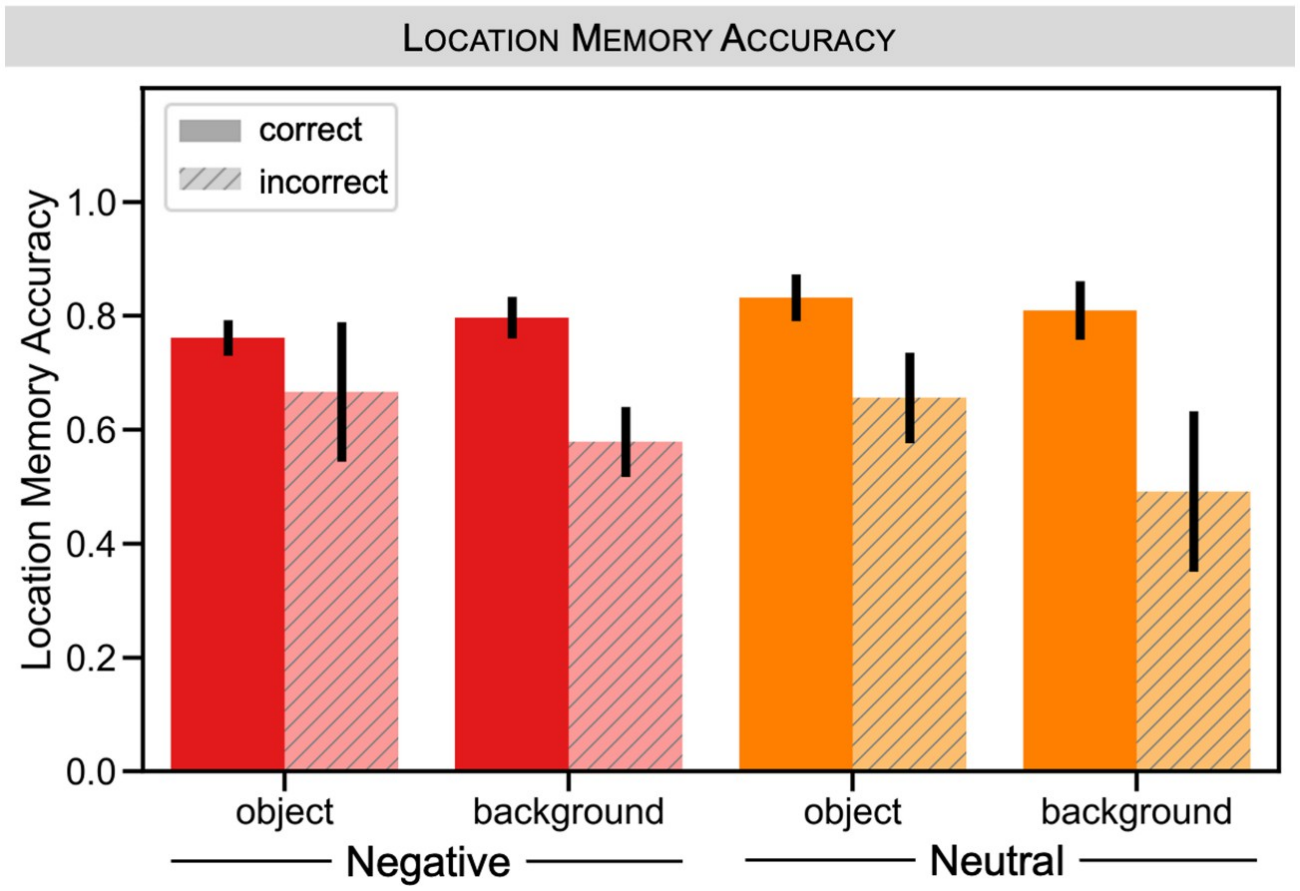
Experiment 2: Location memory by final test memory and object valence. Location memory accuracy is plotted separately for correct (solid) versus incorrect (striped) object and background memory recognition separately for negative (red) and neutral (neutral) trials. All error bars denote standard error of the mean.

Lastly, we examined performance on the location memory task to determine how an explicit measure of location memory (i.e., as opposed to our implicit measure of gaze reinstatement) would correspond to object valence and recognition memory of the scene components (Fig 9). Location memory accuracy was entered into a repeated measures ANOVA with recognition memory (correct, incorrect), scene component (object, background), and object valence (negative, neutral) as within-subjects factors. We had a restricted sample size of 10 participants for this analysis after excluding participants who were missing data in one of our design cells and participants who did not follow instructions on the location task. There was a main effect of recognition memory, *F*(1,9) = 14.931, *p* = 0.004, where location memory was better for trials associated with correct versus incorrect recognition memory. However, there was no main effect of scene component, *F*(1,9) = 1.190, *p* = 0.304, or object valence, *F*(1,9) = 0.006, *p* = 0.939. There was also no interaction between recognition memory and scene component, *F*(1,9) = 1.080, *p* = 0.326, between recognition memory and object valence, *F*(1,9) = 0.775, *p* = 0.402, and scene component and object valence, *F*(1,9) = 1.170, *p* = 0.308. Thus, location memory performance was correlated with performance on the recognition memory tests, but this did not vary by valence or scene component.

### Discussion

In Experiment 2, we corroborated our findings from Experiment 1 and found evidence of stronger gaze reinstatement when object or background memory was correct. This confirms that eye movement patterns are sensitive to the contents of people’s memories. There were more mixed results with regard to the effects of valence on the relationship between gaze reinstatement and memory, suggesting that valence does not strongly moderate the relationship between gaze reinstatement and memory. Finally, in Experiment 2, we additionally showed that explicit measures of location memory were related to recognition memory for the objects and backgrounds, though this did not vary by valence in the limited sample available for this analysis.

## General discussion

*Can we infer people’s memories based on eye movement patterns? And if so, can the eyes also reveal emotional biases that clearly impact memory?* In this pair of experiments, we sought to examine these questions by recording participants’ eye movements while they completed an emotion-induced memory trade-off task. Specifically, we assessed gaze reinstatement during retrieval by whether or not participants looked more at the side of the screen where a negative or neutral object had been presented during encoding.

Across the two studies, we found evidence of more gaze reinstatement on trials associated with correct object or background recognition. This finding extends prior research on memory-related gaze reinstatement [2, 16] by showing that gaze reinstatement is associated with memory benefits for different components of a scene, including a central object as well as its associated background. Prior work has related gaze reinstatement to the accuracy of recognition memory [13, 41], the ability to remember details of specific portions of a scene [15], and the self-reported vividness of recall [14]. These studies have varied in terms of whether they have measured gaze reinstatement during recognition or cued recall. Here we found evidence that gaze reinstatement was consistently related to object and background memory performance during background recognition in both experiments, and that it was related to object memory performance during scene recall in Experiment 2. Because our measure of gaze reinstatement can be interpreted as an implicit measure of memory for the object’s location, it may be that memory for the location helps to cue memory for object and background details. Alternatively, it may be that the strongest memories contain a bound representation of the object’s location as well as the object and scene details that aid recognition. This interpretation is additionally supported by the results from the explicit location memory task in Experiment 2, which showed that explicit object location memory was positively related to recognition memory for both the object and background.

Our central question was whether or not the emotional valence of the object would influence gaze reinstatement. Replicating past work [25], we found strong evidence for an emotion-induced memory trade-off effect [42], such that there was better memory for negative versus neutral objects but at the expense of the associated background memory. Further, during encoding, negative objects attracted more visual attention, as measured by eye movements, than neutral objects. However, we found only limited evidence that valence affected the degree to which these eye movements were reinstated during retrieval. Across the two experiments, gaze reinstatement was consistently related to object and background memory for negative trials, whereas this relationship was often but not always seen for neutral trials. This emerged as a significant valence by memory interaction in both Experiments 1 and 2, although the interaction was observed for object memory in Experiment 1 and background memory in Experiment 2. When considering the data from both experiments together, however, there was not a significant valence by memory interaction for object or background memory, suggesting that valence did not consistently moderate the relationship between gaze reinstatement and memory. Thus, although emotion led to biases that led to better memory for negative objects at the expense of the associated neutral background, it did not significantly affect the way that these memories related to eye movements during retrieval.

What could account for the differences in valence effects between the two experiments? Considering first the experimental designs, the only relevant change was that we decreased the amount of time spent on the retrieval portion of the scene recall phase. In Experiment 1, this portion was 8 s but in Experiment 2 it was 4 s. To see if there were differences in the vividness of scene recall between the two experiments, we examined the vividness ratings participants provided at the end of each scene recall trial and observed no noticeable differences in vividness ratings across the two experiments. Another possibility is that participants in Experiment 1 experienced more study fatigue because they had to keep the associated scenes vividly in mind for twice as long as the participants in Experiment 2, leading to differences in how much gaze reinstatement might be observed across the trial. However, we observed that there was no significant difference between the AUC values produced in the first half of the retrieval trial in Experiment 1 with those produced in the second half. We also analyzed data from self-report questionnaires measuring individual differences in negative affect (PANAS and STAI) to see if they might explain any differences between the two samples but found none (see Section E in S1 Text for more details). Thus, we were unable to identify any specific differences between the samples that might account for the difference in valence results, although we cannot rule out the possibility that such a difference exists.

A limitation of the study is that our sample sizes were relatively small (N = 24 in each study). To address some of the challenges of small sample sizes, we implemented a bootstrapping approach to generate the sampling distribution of AUC values. Many of the reported results replicated across the two studies, instilling confidence in our approach. However, some of the variability in the valence results could have been attributed to random differences between the two samples. For instance, memory performance differences across the two samples could have impacted the number of samples that ended up in each bin (e.g., how many neutral incorrect object recognition trials there were). For instance, comparing the behavioral results plotted in Figs 2 and 6, participants performed worse on the neutral trials in Experiment 2 than Experiment 1, which means that there were more items in the “neutral incorrect” bin when looking for a valence by recognition memory interaction. This could have increased our power to detect an object memory effect for neutral trials in Experiment 2 but not in Experiment 1. Future work examining gaze reinstatement in emotional and neutral contexts would benefit from larger sample sizes, as well as experimental designs that would better balance the number of correct and incorrect memory trials, e.g., by increasing the delay between encoding and retrieval. Another caveat to the current results is that there may have been some differences between negative and neutral objects that made them easier or harder to remember when cued with the scene. This is a common challenge when investigating memory for emotional materials, but could be addressed in future work that explicitly controls for differences between negative and neutral stimuli (e.g., matching the semantic categories in each set).

An innovative aspect of the current study is its experimental design, which allowed for gaze reinstatement to be measured by shifts in gaze toward the left versus right side of the screen. Gaze reinstatement is often measured by taking the correlation of the fixation maps produced at encoding and retrieval. However, this analysis method can be complicated due to individual differences in the dispersion of fixations during imagining versus perception [39, 40]. This motivated us to develop the analysis approach adopted here. By focusing on the differentiation between left and right fixation trials, this approach might also be more sensitive to more nuanced levels of object memory reactivation because it can detect even subtle shifts in gaze behavior. Still, it would be beneficial to investigate how the current approach relates to alternative methods for measuring gaze reinstatement. Because it has been shown that eye movements play in active role in facilitating memory retrieval [16], an interesting future direction would be to examine the causal impact of eye movements on emotional memory retrieval, for instance, by restricting or cuing eye movements, c.f., [43] to encourage or discourage memory retrieval.

In summary, results across two experiments show that backgrounds associated with negative objects reliably elicit a memory-modulated gaze reinstatement effect, demonstrating that memory for the object’s location is intertwined with memory for both the object and background components of the scene. This effect was comparable for negative and neutral memories, which suggests that gaze reinstatement occurs independently of the processes contributing to the emotion-induced memory trade-off effect. To our knowledge, this is the first study investigating gaze reinstatement for emotional and neutral images. The results reported here have the potential to inform future work on how eye movements can be used to detect and influence emotional memory retrieval.

## Supporting information

S1 Text Supplementary materials for “Eye tracking evidence for the reinstatement of emotionally negative and neutral memories”. (PDF)
