# Supplementary material for: Eye tracking evidence for the reinstatement of emotionally negative and neutral memories
Source: PLoS One. 2024 May 17;19(5):e0303755. doi: 10.1371/journal.pone.0303755 (PMC11101026; doi:10.1371/journal.pone.0303755)
Supplement: S1 Text — (PDF) [file pone.0303755.s001.pdf]

Supplementary Materials for
“Eye tracking evidence for the reinstatement of emotionally negative and neutral memories”

Paula P. Brooks, Brigitte A. Guzman, Elizabeth A. Kensinger,
Kenneth A. Norman, and Maureen Ritchey

A. Overview of counterbalancing design

Stimuli for Experiments 1 and 2 consisted of 6 sets of 15 object-background scenes, where each background could be associated with either a negative or neutral object on the left or right side of the scene. Images were assigned to object valence and object location conditions using a Latin-square counterbalancing design that included 6 sets (Table S1).

	Set A	Set B	Set C	Set D	Set E	Set F
Cond. 1	**Negative Left**	Negative Right	Neutral Left	Neutral Right	Neutral Foil	Negative Foil
Cond. 2	Negative Foil	**Negative Left**	Negative Right	Neutral Left	Neutral Right	Neutral Foil
Cond. 3	Neutral Foil	Negative Foil	**Negative Left**	Negative Right	Neutral Left	Neutral Right
Cond. 4	Neutral Right	Neutral Foil	Negative Foil	**Negative Left**	Negative Right	Neutral Left
Cond. 5	Neutral Left	Neutral Right	Neutral Foil	Negative Foil	**Negative Left**	Negative Right
Cond. 6	Negative Right	Neutral Left	Neutral Right	Neutral Foil	Negative Foil	**Negative Left**

Table S1: Latin-Square Counterbalancing Table. Each image set (A-F) had 15 background images that could be paired with a specific object and location in the following conditions: Neutral left, neutral right, negative left, and negative right. The background images and their associated objects could also be foils, appearing only in the recognition memory tests. Participants were randomly assigned into one of these 6 conditions.

B. Analysis of hit and false alarm rates

For both experiments, the emotion-induced memory trade-off effect reported in the main manuscript was evident for hit rates, but not for false alarm rates (Figure S1).

In Experiment 1, hit rate was entered into a repeated measures ANOVA with the object valence (negative, neutral) and scene component (object, background) as within-subjects factors. There was no main effect of object valence, $F(1, 23) = 1.706$, $p = 0.204$, $\eta_p^2 = 0.069$, nor was there a main effect of scene component, $F(1, 23) = 1.800$, $p = 0.193$, $\eta_p^2 = 0.073$. However, there was a significant interaction between object valence and scene component, $F(1, 23) = 46.470$, $p < 0.001$, $\eta_p^2 = 0.669$. There was a higher hit rate for negative objects than for neutral objects, $t(23) = 3.873$, $p = 0.002$, $d_t = 0.791$, but at the expense of the associated background memory. Background memory for associated negative objects was lower than for associated neutral objects, $t(23) = 7.524$, $p < 0.001$, $d_t = 1.536$. False alarm rate was also entered into a repeated measures ANOVA with the object (negative, neutral) and scene component (object, background) as within-subjects factors. There was no main effect of object valence, $F(1, 23) = 2.295$, $p = 0.143$, $\eta_p^2 = 0.091$, nor was there a main effect of scene component, $F(1, 23) = 2.127$, $p = 0.158$, $\eta_p^2 = 0.085$. There was also no significant interaction between object valence and scene component, $F(1, 23) = 0.045$, $p = 0.834$, $\eta_p^2 = 0.002$. There was no significant difference in false alarm rate between negative and neutral objects, $t(23) = 0.861$, $p = 0.796$, $d_t = 0.176$, nor was there a significant difference between negative and neutral backgrounds, $t(23) = 1.282$, $p = 0.425$, $d_t = 0.262$.

In Experiment 2, hit rate was entered into a repeated measures ANOVA with the object (negative, neutral) and scene component (object, background) as within-subjects factors. There was no main effect of object valence, $F(1, 23) = 0.205$, $p = 0.655$, $\eta_p^2 = 0.008$, nor was there a main effect of scene component, $F(1, 23) = 1.266$, $p = 0.272$, $\eta_p^2 = 0.052$. However, there was a significant interaction between object valence and scene component, $F(1, 23) = 44.170$, $p < 0.001$, $\eta_p^2 = 0.658$. There was a higher hit rate for negative objects than for neutral objects, $t(23) = 5.203$, $p < 0.001$, $d_t = 1.062$, but at the expense of the associated background memory. Background

memory for associated negative objects was lower than for associated neutral objects, $t(23) = 5.414$, $p < 0.001$, $d_t = 1.105$. False alarm rate was also entered into a repeated measures ANOVA with the object (negative, neutral) and scene component (object, background) as within-subjects factors. There was a main effect of object valence, $F(1, 23) = 8.245$, $p = 0.009$, $\eta_p^2 = 0.264$ but there was no main effect of scene component, $F(1, 23) = 0.439$, $p = 0.514$, $\eta_p^2 = 0.019$, nor was there a significant interaction between object valence and scene component, $F(1, 23) = 1.000$, $p = 0.328$, $\eta_p^2 = 0.042$. There was a significant difference in false alarm rate between negative and neutral objects, $t(23) = 2.716$, $p = 0.025$, $d_t = 0.554$, but there was no significant difference between negative and neutral backgrounds, $t(23) = 1.238$, $p = 0.456$, $d_t = 0.253$.

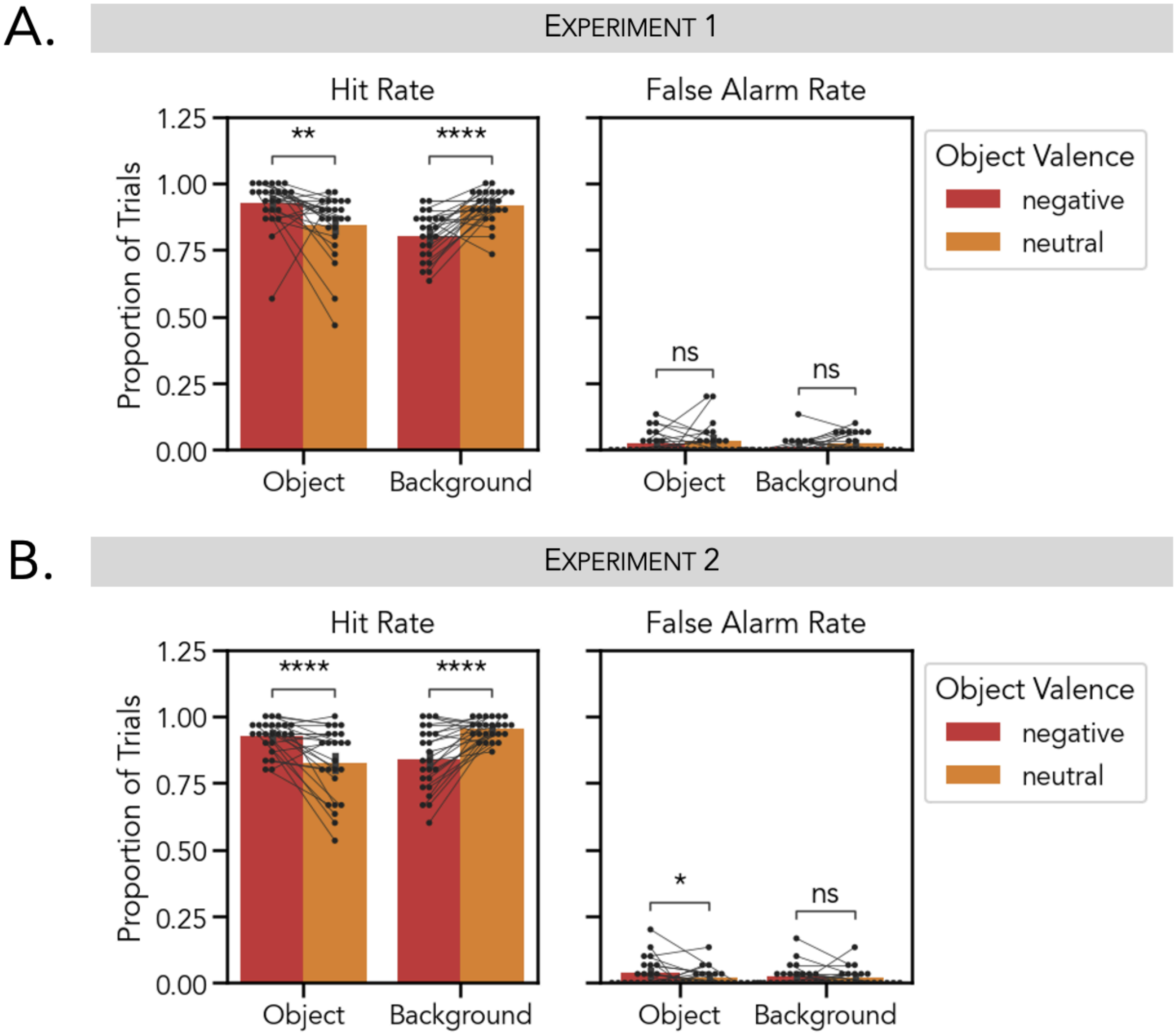

Figure S1: Final Test Performance by Hit and False Alarm Rates. We looked at final test performance by hit and false alarm rates. For both (A) Experiment 1 and (B) Experiment 2, there was a significant interaction between object valence and scene component for hit rate but not false alarm rate. The hit rate was higher for negative objects than for neutral objects but at the expense of the associated background. All error bars denote standard error of the mean. Paired comparisons were performed using paired t-tests with Bonferroni correction, * $p < 0.05$, ** $p < 0.01$, **** $p < 0.0001$.

C. Effect of object location on the emotional memory trade-off effect

For the behavioral results, we wanted to ascertain whether the emotion-induced memory trade-off effect was affected by a memory bias towards objects encoded in the left or the right object location (Figure S2). As a result, we also looked at hit rate by object location along with object valence and image type.

In Experiment 1, we added object location (left, right) to the within-subjects factors in the repeated measures ANOVA we described in the previous section, where hit rate was entered in the ANOVA with the object valence (negative, neutral) and scene component (object, background) as within-subjects factors. There was no main effect of object location $F(1, 23) = 2.104$, $p = 0.160$, $\eta^2_G = 0.007$. There were also no significant interactions between scene component and object location, $F(1, 23) = 0.363$, $p = 0.553$, $\eta^2_G = 0.001$, between object valence and object location, $F(1, 23) < 0.001$, $p = 1.000$, $\eta^2_G < 0.001$, or between all three factors, $F(1, 23) = 0.070$, $p = 0.794$, $\eta^2_G < 0.001$.

In Experiment 2, we added object location (left, right) to the within-subjects factors in the repeated measures ANOVA we described in the previous section. There was no main effect of object location $F(1, 23) = 0.160$, $p = 0.693$, $\eta^2_G < 0.001$. There were also no significant interactions between object valence and object location, $F(1, 23) = 0.663$, $p = 0.423$, $\eta^2_G = 0.001$, or between scene component, object valence, and object location, $F(1, 23) = 0.250$, $p = 0.622$, $\eta^2_G = 0.001$. On the other hand, there was a significant interaction between scene component and object location,

$F(1, 23) = 12.766$, $p = 0.002$, $\eta^2_G = 0.018$. However, there was no significant difference in object memory between left and right object location, $t(23) = 1.579$, $p = 0.121$, $d_t = 0.322$, nor was there a significant difference in background memory between left and right object location, $t(23) = 1.829$, $p = 0.074$, $d_t = 0.373$. Thus, although there was a significant interaction, there was not a significant effect of object location on either scene component. All in all, we did not find a bias in hit rate towards the left or the right location.

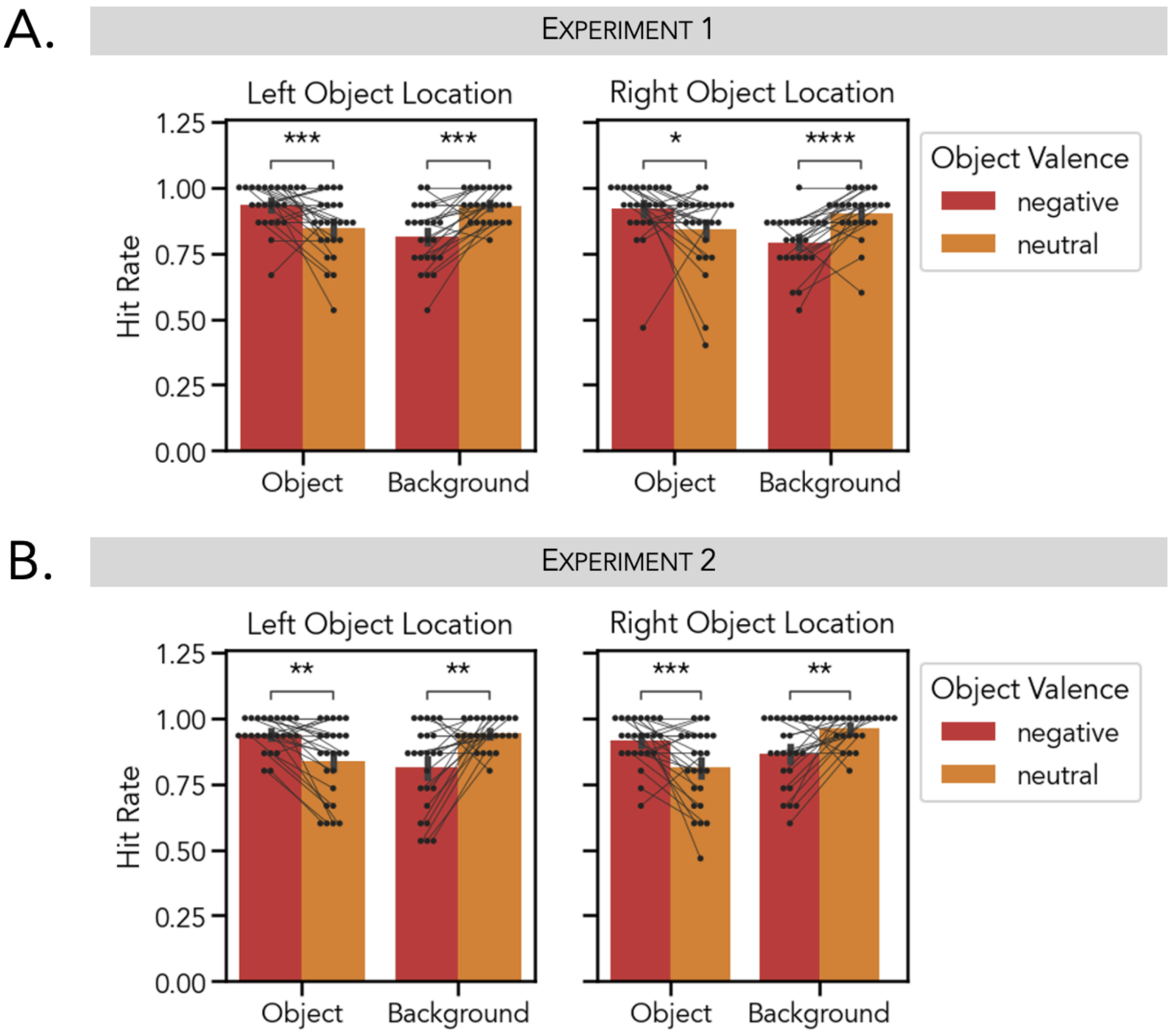

Figure S2: Hit Rate by Object Location, Object Valence, and Image Type. We looked at hit rate by object location, object valence, and image type. For both (A) Experiment 1 and (B)

Experiment 2, there was evidence of the emotion-induced memory trade-off effect—where hit rate was higher for negative objects than for neutral objects but at the expense of the associated background—regardless of whether or not the object appeared on the left or the right. All error bars denote standard error of the mean. Paired comparisons were performed using paired t-tests with Bonferroni correction, *$p < 0.05$, ** $p < 0.01$, *** $p < 0.001$, **** $p < 0.0001$.

D. Gaze reinstatement analysis combining data from Experiments 1 and 2

We ran a follow-up analysis using the combined data from Experiments 1 and 2 (total $N = 47$) to look at the interaction between memory and valence in the gaze reinstatement during the background recognition phase (Figure S3). To conduct this analysis, we resampled the data with replacement from Experiments 1 and 2 separately before aggregating all the data to compute an AUC value to measure gaze reinstatement, as in our main analyses. We did this bootstrapping procedure 1000 times.

For negative valence items, we found that AUC was modulated by object and background memory. There was a larger AUC value for correct versus incorrect negative object memory (mean difference = 0.233, $p = 0.001$). We found a similar pattern for background memory (mean difference = 0.134, $p = 0.002$). However, AUC was modulated by object memory but not for background memory for neutral valence items. There was a larger AUC value for correct versus incorrect negative object memory (mean difference = 0.137, $p = 0.001$). However, there was no significant difference in AUC values for correct versus incorrect neutral background memory (mean difference = 0.020, $p = 0.404$). To quantify the interaction between memory performance and valence condition, we computed the AUC differences separately for object and background memory. Specifically, we computed the difference between the AUC scores during background recognition for trials associated with correct versus incorrect object or background memory separately for our two valence conditions; and we then computed the difference between negative versus neutral. We did not find a significant interaction effect for object memory, $p = 0.109$, or for background memory, $p = 0.112$.

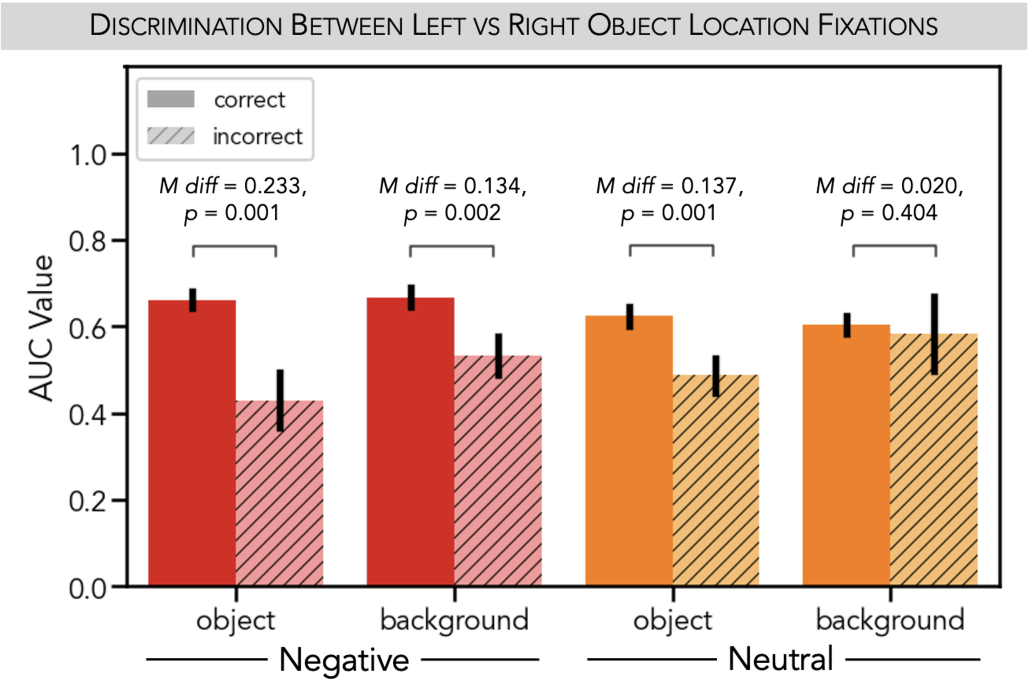

Figure S3: Gaze reinstatement results using combined data and split by valence and memory. We looked at the separability of fixations associated with left versus right object locations during background recognition by object valence and final test performance on the combined data from Experiments 1 and 2. The figure depicts AUC values that quantify the separation of the weighted average values for left versus right object trials following our bootstrapping procedure, computed separately for negative (red) and neutral (orange) trials when the participant got the object and background correct (solid) versus incorrect (striped). The reported statistics correspond to the difference in AUC values between correct versus incorrect object or background memory. All error bars denote standard deviation of the bootstrap.

E. Analysis of self-reported questionnaires

Finally, we ran a follow-up analysis to see if there were any differences in responses to the self-reported questionnaires (PANAS and STAI) between the participants who completed Experiment 1 versus those who completed Experiment 2 (Figure S4). Participants with missing data in a questionnaire were excluded from analysis. We compared the questionnaire subscale scores for participants in Experiment 1 versus those in Experiment 2 using independent t-tests.

There were no significant differences between the two samples across the subscales of the two questionnaires: the PANAS positive affect score, $t(45) = 1.697$, $p = 0.0965$; the PANAS negative affect score, $t(45) = 0.691$, $p = 0.493$; the STAI state anxiety score, $t(41) = 0.007$, $p = 0.994$; the STAI trait anxiety score, $t(41) = 1.507$, $p = 0.139$.

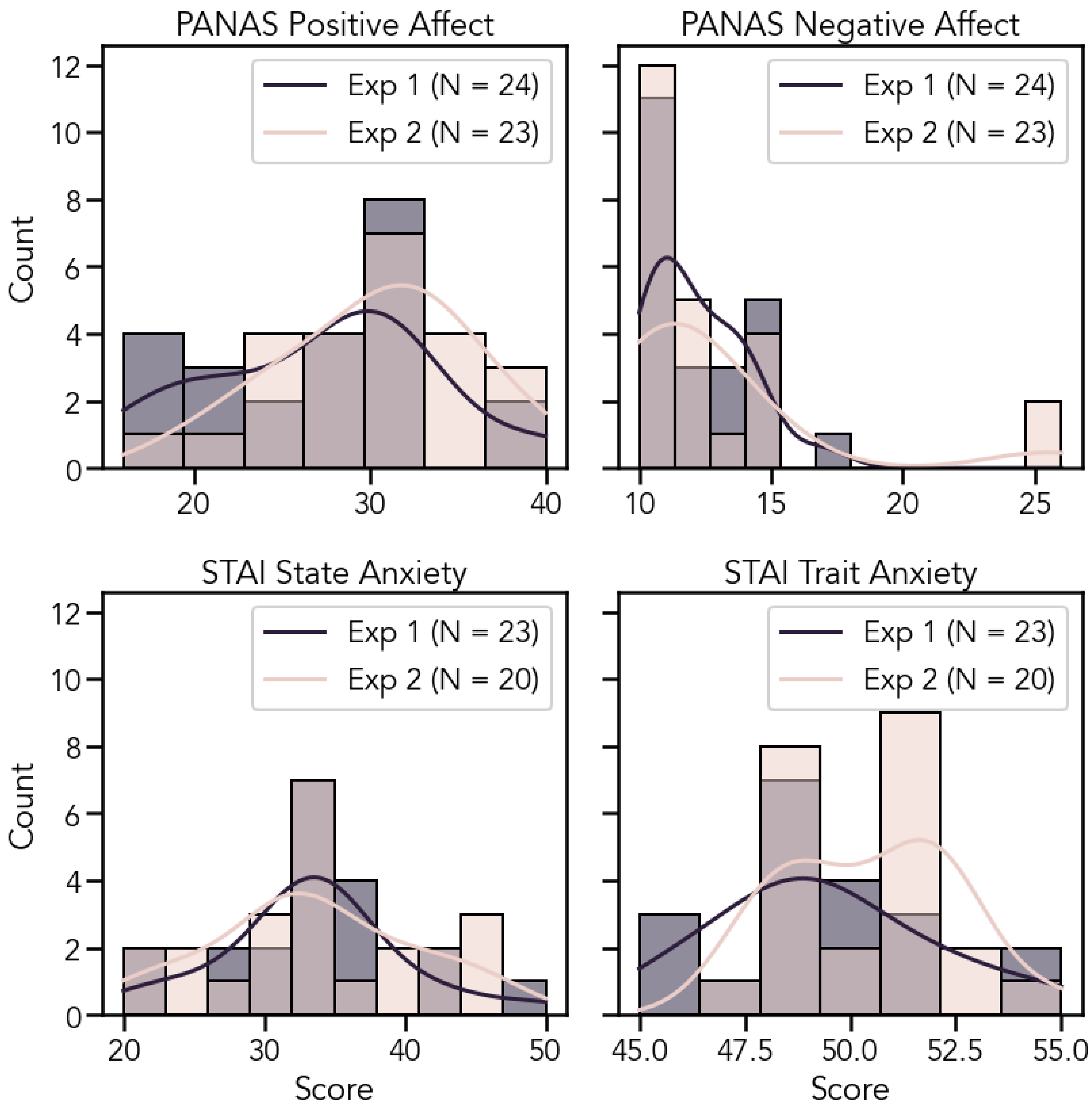

Figure S4: Self-reported questionnaire responses for Experiments 1 and 2. We compared the responses between the two samples on the self-reported questionnaires. We found no differences in positive or negative affect, as reported on the PANAS questionnaire.

We also did not find any differences in state or trait anxiety, as reported on the STAI questionnaire.
